# Assessment of Movement Patterns during Intubation between Novice and Experienced Providers Using Mobile Sensors: A Preliminary, Proof of Concept Study

**DOI:** 10.1155/2015/843078

**Published:** 2015-06-16

**Authors:** Jestin N. Carlson, Samarjit Das, Stephanie Spring, Adam Frisch, Fernando De la Torre, Jessica Hodgins

**Affiliations:** ^1^Department of Emergency Medicine, Allegheny Health Network, Erie, PA 16544, USA; ^2^The Robotics Institute, Carnegie Mellon University, Pittsburgh, PA 15213, USA; ^3^Department of Emergency Medicine, Albany Medical Center, Albany, NY 12208, USA

## Abstract

*Background.* There are likely marked differences in endotracheal intubation (ETI) techniques between novice and experienced providers. We performed a proof of concept study to determine if portable motion technology could identify the motion components of ETI between novice and experienced providers.* Methods.* We recruited a sample of novice and experienced providers to perform ETIs on a cadaver. Their movements during ETI were recorded with inertial measurement units (IMUs) on the left wrist. The signals were assessed visually between novice and experienced providers to identify areas of differences at key steps during ETI. We then calculated spectral smoothness (SS), a quantitative measure inversely related to movement variability, for all ETI attempts.* Results.* We enrolled five novice and five experienced providers. When visually inspecting the data, we noted maximum variability when inserting the blade of the laryngoscope into the mouth and while visualizing the glottic opening. Novice providers also had greater overall variability in their movement patterns (SS novice 6.4 versus SS experienced 26.6).* Conclusion.* Portable IMUs can be used to detect differences in movement patterns between novice and experienced providers in cadavers. Future ETI educational efforts may utilize portable IMUs to help accelerate the learning curve of novice providers.

## 1. Introduction

Endotracheal intubation (ETI) is an advanced airway procedure that is defined by a series of movements that result in a tube passing through the glottic opening into the trachea to allow for oxygenation and ventilation. Unsuccessful or prolonged ETI efforts can lead to multiple complications including hypoxia, brain damage, and even death [1–3]. These complications may be magnified when performing ETI in acute care settings including the emergency department and out-of-hospital environments [1, 3, 4]. As a result, learning ETI in acute care settings is challenging and often the learning curve for ETI in these settings is prolonged [5, 6]. Procedural competency is essential for low-frequency and high-consequence procedures such as ETI and therefore it is essential to accelerate the learning curve for emergent ETI.

While there are educational programs for teaching ETI, there are few objective metrics available to assess procedural competency, specifically the kinematics involved in ETI. An improved understanding of the motions involved in ETI and their connections with airway exposure and visualization could impact airway education practices, shedding light on the unrecognized actions needed to accomplish ETI and improve patient outcomes. Previous work with motion capture has identified differences in movement patterns between novice and experienced providers [7]. This work has been restricted to mannequin models due to limited portability of motion analysis technology. Portable inertial measurement units (IMUs) have been used in other clinical settings, but their utility in ETI is unknown [8, 9].

We demonstrate proof of concept that ETI motions can be recorded by portable IMUs. We hypothesize that portable IMUs can identify movement patterns that differentiate novice from experienced providers when performing ETI outside of mannequins.

## 2. Methods

### 2.1. Study Design and Setting

We performed an interventional, observational study examining the movement patterns of providers while performing intubation on a cadaver. After providing informed consent, participants were outfitted with IMUs (Emerald Model, APDM Inc, Portland, OR) on the left wrist. Participants then performed one intubation attempt on a cadaver using the CMAC video laryngoscope (Model 8402, Karl Storz Corp., Tuttlingen, Germany) with a #4 Macintosh blade. Participants used the CMAC as a direct laryngoscope; however, their ETI attempts were recorded for offline review. All intubation attempts were made with the cadaver on the anatomy table (fixed height of 89.5 cm). The providers' movements were recorded with the IMUs. Prior to each ETI attempt, providers were instructed to clap three times, pick up the laryngoscope with their left hand, move the laryngoscope up and down three times, lay the laryngoscope back down, and rest their hands on the table. This provided a unique signal, allowing us to synchronize the videos from the CMAC and the movement patterns from the IMUs to identify the beginning of the intubation attempt. Providers' movements, as recorded by the IMUs, were then compared offline between experienced and novice providers. This study was approved by our Institutional Review Board.

### 2.2. Selection of Participants

We recruited a convenience sample of five providers from a pool of attending physicians and fourth year emergency medicine residents with each having over 100 ETIs in the clinical setting, and defined these as “experienced” providers. We also recruited a convenience sample of five third and fourth year medical students with each having <10 ETIs in the clinical setting and defined these as “novice” providers. All providers had previous formal airway training. We defined “previous formal airway training” as having attended a structured airway didactics of ≥1 hour in length for experienced providers (emergency medicine resident or attending physician). Novice providers must have attended a structured airway didactics of ≥1 hour in length or completed a rotation in anesthesia. No participants reported significant experience with the CMAC prior to this study. We excluded providers who had performed between 10 and 100 ETIs or if they had no formal airway training.

### 2.3. Cadaver Preparation

All intubations were made on a single, embalmed, male human cadaver. The cadaver had no oral, pharyngeal, or neck trauma, craniofacial abnormality, or a known history of tracheostomy. We recorded anatomic measurements related to airway placement including thyromental distance (6 cm), thyrohyoid distance (2 cm), and neck circumference (56 cm) at the level of the thyroid cartilage [10]. Initially, a 2-inch incision was made through the skin over the area of the zygomatic arch down towards the jaw line. The skin was reflected inferiorly to expose the underlying structures. The parotid gland and subcutaneous tissues were removed in order to expose the masseter and its origins on the zygomatic arch. The superficial and deep heads of the masseter were detached from the zygomatic arch and retracted inferiorly in order to expose the mandible. In order to permit more free motion of the jaw, the temporalis muscle was then detached from its insertion on the coronoid process of the mandible. This allowed providers to instrument anatomic structures during ETI attempts and created a grade 3 Cormack-Lehane view as assessed by the investigators. We created a grade 3 Cormack-Lehane view as we felt this would allow for greater discrimination between the movement patterns of novice and experienced providers.

### 2.4. Methods and Measurements

We collected provider demographics and recorded both the movement patterns of the IMUs using the IMU integrated software along with video of the intubation attempt using the integrated CMAC software. Placement of the endotracheal tube (trachea versus esophagus) was assessed by visual inspection by the investigators after each ETI attempt. We defined an intubation attempt each time the blade of the laryngoscope entered the mouth. We defined attempt time as the time in seconds from when the blade of the laryngoscope entered the mouth until it was fully withdrawn from the mouth after placement of the endotracheal tube (either successful placement in the trachea or unsuccessful in the esophagus).

### 2.5. Outcomes

Our primary outcome was variability in movement patterns assessed by spectral smoothness and visual accelerometer patterns between novice and experienced providers during ETI.

### 2.6. Analysis

**Figure 1:**
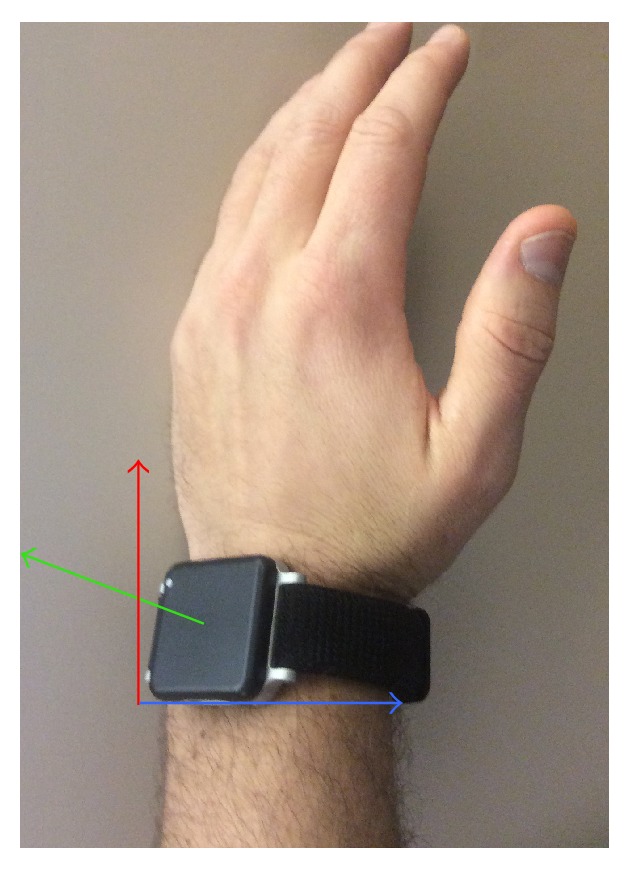
Inertial measurement unit on the left wrist. Blue arrow, *x*-axis. Red arrow, *y*-axis. Green arrow, *z*-axis.

Providers had their movements recorded during ETI using an IMU placed on the posterior aspect of the left wrist. We collected accelerometer data in the *x*-, *y*- and *z*-axes (Figure 1). We visualized the data to identify the IMU and axis with maximum variability and utilized these data for analysis. We segmented the IMU data into portions of the ETI attempt based on previous ETI motion analysis: laryngoscope entering the mouth, obtaining the view of the vocal cords, placing the endotracheal tube, and removing the laryngoscope [7]. We computed 16-point Fast Fourier Transform (FFT) on the segmented signal [11, 12]. A quantitative measure of spectral smoothness (SS) was computed over all trials corresponding to each group (novice and experienced). The SS measure was computed from the FFT of accelerometer data as follows [13]:

1. *X* is the FFT vector;
2. SS = *σ*(*δ*[*X*])/‖*m*(*δ*[*X*])‖, where ‖·‖ is the absolute value, *σ*(·) is the standard deviation, *δ*[·] is first order differential, and *m*(·) is the mean function.

SS is a nonnegative (>0) measure, which is inversely proportional to the absolute value of the mean of signal differential. Therefore, the choppier the signal (e.g., greater variability or less smooth), the lower the SS value. This is due to larger differences in successive signal samples and thus high absolute value of the mean of differential signal. A smooth signal results in low absolute mean value of the differential signal, thereby increasing the SS value. Thus, the SS measure represents the extent of redundant motion variations (i.e., choppiness) associated with ETI attempts [13]. These computations were completed with MATLAB, release 2012b (version 8.0, MathWorks, Inc, Natick, MA).

We also sought to identify patterns that may exist in the movement signals between novice and experienced providers. Movement patterns consist of two aspects, the dispersion or distance traveled and the acceleration or quickness. Dispersion in the *x*-, *y*-, and *z*-axes is not simple straight lines, but it represents complex mathematical signals. As a result, dispersion is often difficult to represent as a single sinusoidal equation. However, each ETI dispersion signal can be represented as a collection of simpler sinusoidal waves with different frequencies (as measured in hertz or Hz) that combine to form the overall sinusoidal equation. The second movement component, acceleration, can be measured by the accelerometers in the IMUs, and expressed as force (in gravitational constants or *g*). To directly compare the overall movement patterns between novice and experienced providers, we graphed the net value of force, in *g* ^2^, by the various simpler sinusoidal frequencies that constitute the overall complex sinusoidal equation for dispersion [11, 12]. Based on the computed 16-point Fast Fourier Transform (FFT) of the overall complex signal, the components can be equally transformed into 16 discrete units representing simpler sinusoidal waves between −60 Hz and 60 Hz. (11, 12) This allowed us to visually compare the movement components, both dispersion and force, between experienced and novice providers.

## 3. Results

**Figure 2:**
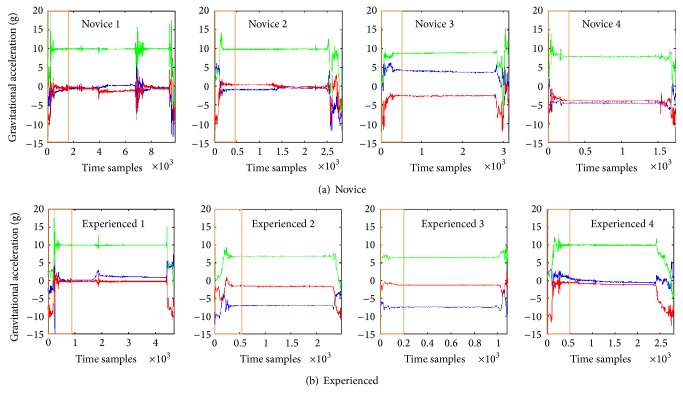
Complete movement patterns in the *x*-, *y*-, and *z*-axes for the four novice (a) and experienced (b) providers. The blue line represents movement in the *x*-axis. The red line represents movement in the *y*-axis. The green line represents movement in the *z*-axis. The orange box represents the laryngoscope entering the mouth and obtaining the view of the vocal cords (magnified in Figure 3).

**Figure 3:**
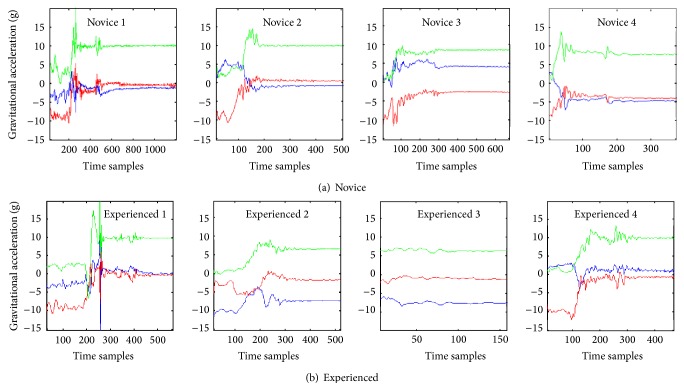
Complete movement patterns in the *x*-, *y*-, and *z*-axes for the four novice (a) and experienced (b) providers from blade insertion until glottic visualization. The blue line represents movement in the *x*-axis. The red line represents movement in the *y*-axis. The green line represents movement in the *z*-axis.

We enrolled five novice (one third year and four fourth year medical students), and five experienced providers (four fourth year emergency medicine residents and one attending physician) (Table 1). Due to troubles with recording, one provider in each group did not have their IMU signal available for analysis and was thus excluded from the study. The IMU data corresponding to the laryngoscope insertion and glottis visualization was then segmented out for further analysis (Figure 2). We compared the IMU data to the videos recorded during the intubation attempt and identified the four segments of the kinematic signals: laryngoscope entering the mouth, obtaining the view of the vocal cords, placing the endotracheal tube, and removing the laryngoscope [7]. The orange box (Figure 2) represents the steps where the laryngoscope entered the mouth and a view of the vocal cords was obtained. Visually, there appeared to be the greatest movement variability during this step; thus, this area is magnified in Figure 3.

**Figure 4:**
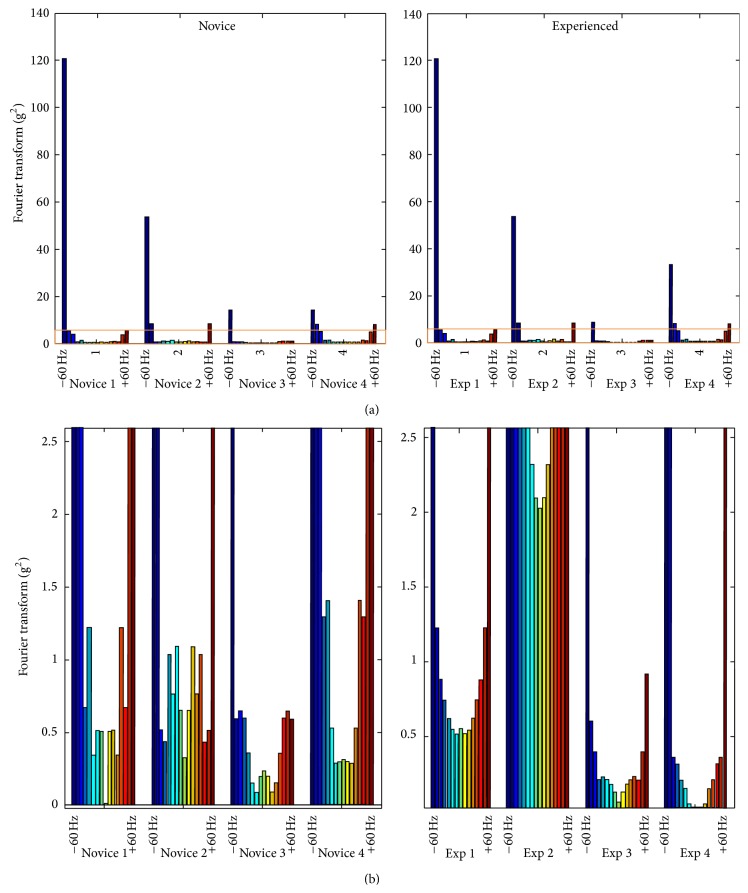
Spectral analysis of the *Z*-component of IMU signal from insertion until glottic visualization for the four novice and experienced providers. Spectral range (blue to red) for each subject is ±60 Hz divided into 16 equal segments. The overall signals are shown in the top graphs. The orange box represents the areas magnified in the lower graphs. Exp: experienced.

After transforming the data via the FFT, the spectral analysis of the *Z*-component from these sections had a parabolic curve for both novice and experienced providers (Figure 4). This curve was smoother for experienced providers both on visual inspection and when analyzed by spectral smoothness (SS novice 6.4 versus SS experienced 26.6).

When the complex movement patterns in the *z*-axis were broken down into simpler frequencies, there appeared to be a unique, parabolic relationship between these sinusoidal waves (the description of movement) and force with experienced providers (Figure 4). Experienced providers had a bimodal distribution of forces, where greater forces were noted at lower frequency signals and then again at higher frequency signals.

## 4. Limitations

There are several limitations to this study. Our study was limited to a small sample. Initially, we sought to compare five novice and five experienced providers but had to limit the study size to four providers in each group due to incomplete recording of the data. Second, this study was performed in a cadaver model with a difficult airway (grade 3 Cormack-Lehane). The cadaver underwent a modified dissection of tissue, masseter, and temporalis muscle detachment to allow for a grade 3 Cormack-Lehane view. We felt a difficult airway (Cormack-Lehane grade 3) would allow for greater discrimination between the movement patterns of novice and experienced providers. As Cormack-Lehane grade 3 views are infrequently encountered in emergency airway management, we focused our efforts on the cadaver model [14–16]. Also, from a patient safety and research ethics standpoints, we did not feel it was in patients' best interest to perform multiple intubation attempts on a patient or patients with a difficult airway, especially with novice providers, for a proof of concept study. The cadaver allowed us to standardize the intubation attempts as all attempts could then be made on one airway. While we are able to show proof of concept that IMUs are able to collect data and differentiate between novice and experienced providers in ETI using human tissue, future efforts will be needed to assess IMUs in the clinical setting with varying glottic views. Studies involving comparison between cadaver and human subjects would provide further insight with the kinematics involved in ETI. Other movement patterns that may be critical to ETI success were not investigated. As we were only able to analyze left wrist movement patterns, future investigations may examine other joints (elbow, shoulder, right wrist, etc.) to provide a more complete analysis of the kinematics involved in ETI.

## 5. Discussion

We were able to identify differences between experienced and novice providers using IMUs in a cadaver model. By identifying these key differences, future work may provide further quantification and impactful feedback during ETI instruction. Eventually, this may be incorporated with a model that allows real-time feedback via instruction and correction while performing the task of ETI in a clinical setting. ETI proficiency is associated with procedural experience with this skill [6]. If we are able to further accelerate the learning curve of providers, competency may be achieved at a faster rate, thus reducing the potential harmful of the learning curve.

In a multicenter analysis including over 6,000 ETIs, the first attempt success rates varied by provider experience with the first year emergency medicine residents having success rate of only 72% compared to 82% and 88% in the second year and third year, respectively [17]. First attempt success also decreases with Cormack-Lehane view where grade 3 views have first attempt success rates near 40%, similar to those noted in our study [16]. Complications occur more frequently in cases where multiple ETI attempts are made [3]. Accelerating the learning could directly address the complications related to multiple attempts in novice providers in the acute setting.

We chose to evaluate the movements of the left wrist in novice and experienced providers during intubation. While there is not yet a clear link between wrist movements and intubation success or side effects, there are distinct differences in the movement patterns of the left wrist between novice and experienced providers [7]. As providers gain experience with emergency airway management, they demonstrate greater intubation success and lower rates of complication related to intubation [17, 18]. Examining the link between these movement patterns and intubation outcomes may provide insight into why these differences in intubation success exist and identify opportunities for improvement in ETI techniques.

To our knowledge, ours is the first study using portable movement mapping technology to evaluate intubation. The benefits of portable sensors have yet to be fully realized in the acute care setting. Portable sensors may not be limited to the use of IMUs but may also make use of other technologies such as smartphones with incorporated cameras and accelerometers. Previous work has shown that smartphones may help with ETI and can even monitor chest compression during cardiopulmonary resuscitation [19–21]. The ubiquitous nature of the technologies incorporated into smartphones represents an ideal tool for capturing information and providing feedback.

While our study was designed as a proof of concept, we believe that portable sensors may be able to identify movement patterns between providers with different levels of experience. These technologies can identify patterns of force that may vary with different components of the overall dispersion signal (i.e., there might be dispersion differences between not only novice and experienced providers measured in space, but also the force with which these actions take place). Despite our small sample size, there appear to be differences in the combination of dispersion and force between novice and experienced providers where experienced providers had a bimodal distribution of forces, where greater forces were noted at lower frequency signals and then again at higher frequency signals while this was not seen with novice providers (Figure 4).

Interpreting these findings can be challenging but may be contextualized more easily using an example outside of medicine. When assessing how someone may swing a golf club, there are two components to the swing, the dispersion (or measurement of the distance moved) and the acceleration or force. The golfer needs the ideal “mechanics” or dispersion combined with the proper force at the correct time during the swing. Representing the relationship between the dispersion and force allows for the identification of key differences within the movement pattern. Identifying the unique interaction between dispersion and force may also help to explain the differences in ETI success rates between providers and allow for focused feedback to novice providers.

## 6. Future Directions

The clinical implications of this line of work are broad. Future work with portable sensors may help to track the movements of novice providers, compare these movements to those of experienced providers, and provide real-time, objective feedback to trainees on their movement patterns. While we have focused on ETI, similar educational models could be developed for other medical procedures. The successful development of these models requires multiple steps:

1. identify portable sensors that can objectively track movement patterns in the clinical setting;
2. classify movement patterns that differentiate novice from experienced providers;
3. incorporate analysis algorithms that will allow for rapid assessment of movement patterns and recognize areas that require focused educational attention (e.g., what portion of the ETI attempt differed from that of previously analyzed experienced ETI attempts);
4. develop teaching curriculum that incorporates these measurements to allow for impactful, timely feedback.

While we have shown proof of concept that ETI motions can be recorded by portable IMUs (step (1)), future work will be needed to effectively develop this technology into an educational modality (steps (2)–(4)).

Ericsson's model of deliberate practice presents a framework for this information to be leveraged [22, 23]. Ericsson states that deliberate practice must provide immediate feedback, correction, remediation, and repetition [6, 22, 23]. The inclusion of additional feedback to the student from the kinematic data allows for more precise and immediate feedback beyond a simple yes/no of success with the performance of ETI. While kinematic feedback may require a better understanding of the whole body movements of the provider during ETI, we chose to focus our preliminary efforts on the left wrist as there are distinct differences in the movement patterns of the left wrist between novice and experienced providers [7]. Experienced providers also have greater intubation success and lower rates of complication indicating a potential link between movement patterns and intubation success [17, 18]. A more nuanced understanding of the entire ETI process presents additional opportunity for the practitioner to receive immediate feedback on these movement differences and accelerate the learning curve.

Specific to simulation and mannequin based learning, prior studies including Hall et al. have also shown increased skill acquisition with the combination of simulated and mannequin based training [24]. With the additional data gleaned from a more complex mapping of the novice versus experienced movements made during ETI, we may further enhance skill acquisition outside of clinical practice. Segmenting the steps and breaking down the process of ETI may also allow for more precise practice with cadavers and mannequins.

Movement sensor analysis provides valuable, objective data and has been used in a variety of clinical settings. Other studies have used portable sensors to track progression after stroke [8, 9]. This line of work has shown that motion sensor analysis presents a linear relationship with subjective measures of stroke severity. In a similar manner, motion sensor analysis in the use of ETI may provide a more objective measure of techniques utilized in successful ETI beyond subjective feedback of an instruction practitioner. Future efforts are needed to advance the kinematic analysis process and provide subjects with real-time feedback.

## 7. Conclusion

IMUs can be used to identify the kinematics of both novice and experienced providers in a cadaver model. By further understanding movement patterns for ETI and quantitatively analyzing ETI kinematics, we are better able to understand the mechanics of intubation. These are the first steps in designing a real-time feedback system to accelerate the learning curve of ETI.

## Figures and Tables

**Table 1:** Provider characteristics. EM: emergency medicine.

	Novice (*n* = 5)	Experienced (*n* = 5)
Age in years, (SD)	27 (2.8)	31.4 (1.3)
Sex: male (*n*)	80% (4)	80% (4)
Handedness: right	100% (5)	100% (5)
Experience	Fourth year medical student: 4 Third year medical student: 1	Fourth year EM resident: 4 EM attending: 1
First attempt success (*n*)	20% (1)	40% (2)
Attempt time in seconds (mean, SD)	38.3 (9)	33 (5.5)
